# Supplementary material for: Potential of training of anti-Staphylococcus aureus therapeutic phages against Staphylococcus epidermidis multidrug-resistant isolates is restricted by inter- and intra-sequence type specificity
Source: mSystems. 2024 Sep 9;9(10):e00850-24. doi: 10.1128/msystems.00850-24 (PMC11494967; doi:10.1128/msystems.00850-24)
Supplement: Supplemental Figures — Figures S1 to S3. [file msystems.00850-24-s0001.docx]

**Supplementary Figure S1. Bacterial growth inhibition of S. epidermidis ST2 strains by trained phage E1 in comparison to ancestral phages.**

Each dot represents the mean Liquid Assay Score (LAS) for each ST2 strain obtained from experiments performed in triplicates.


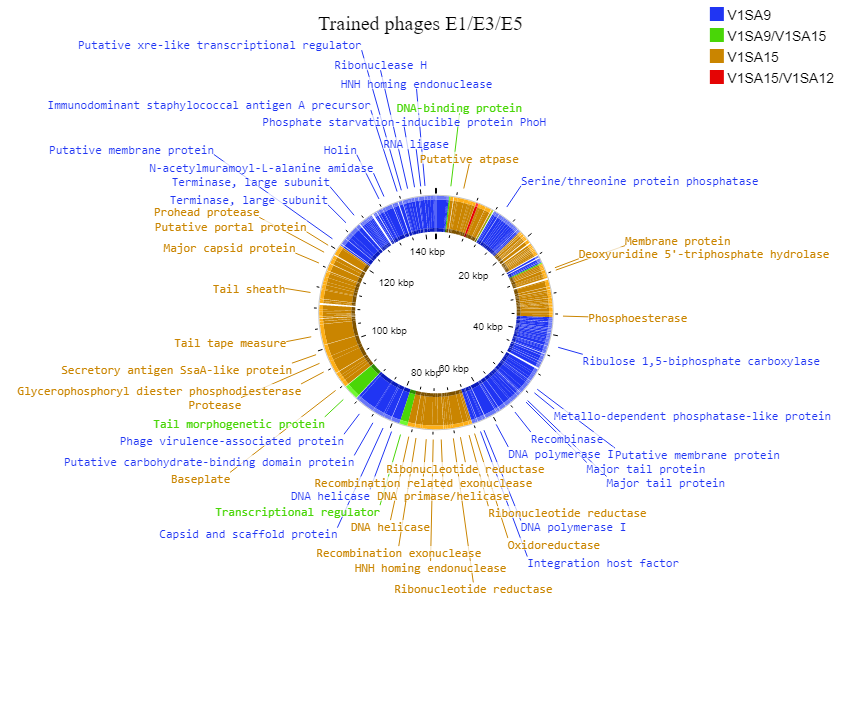


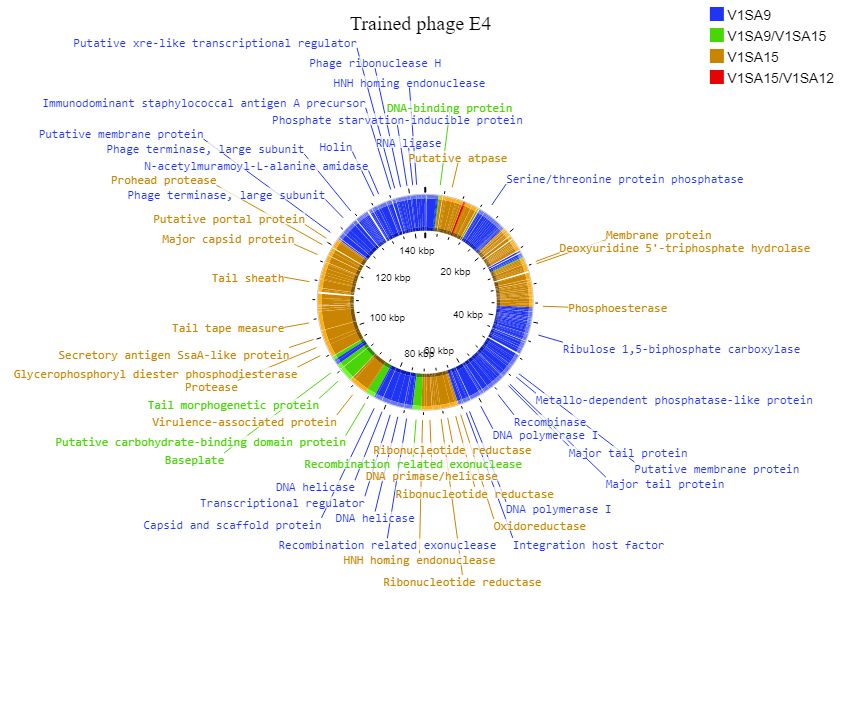
**A**


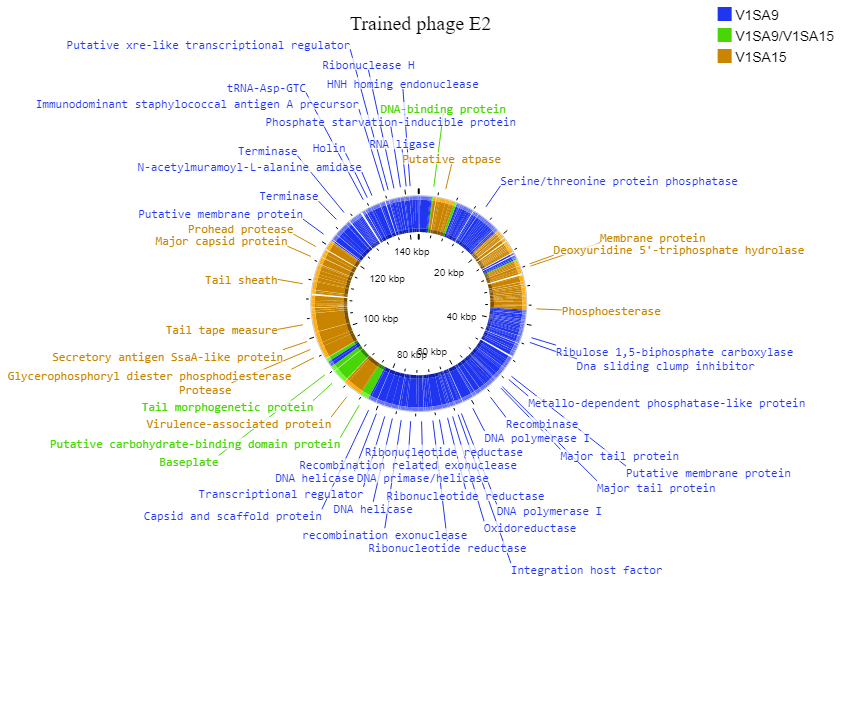


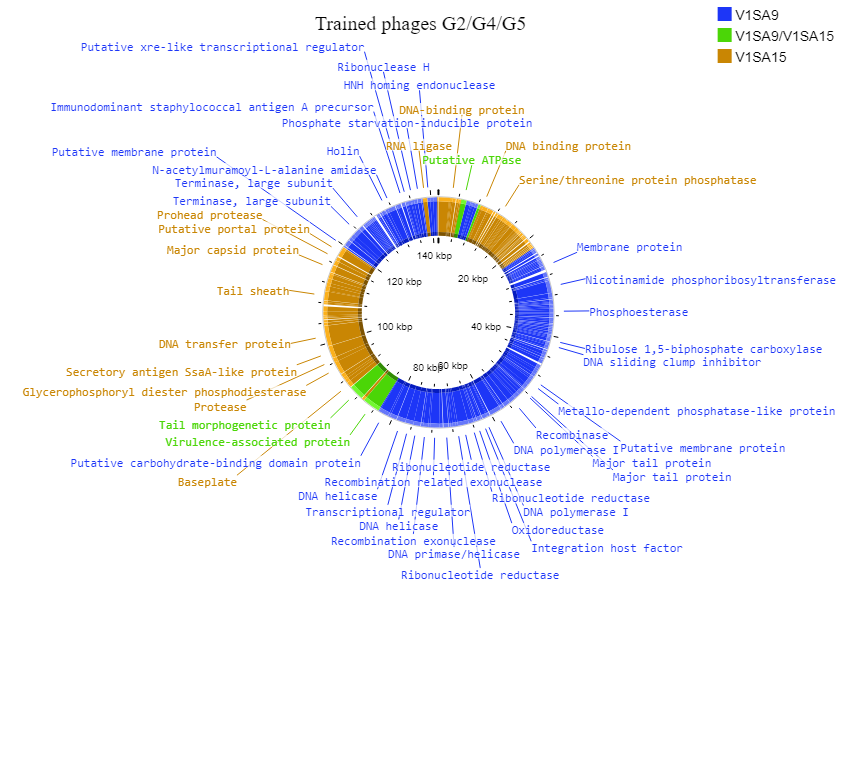
**
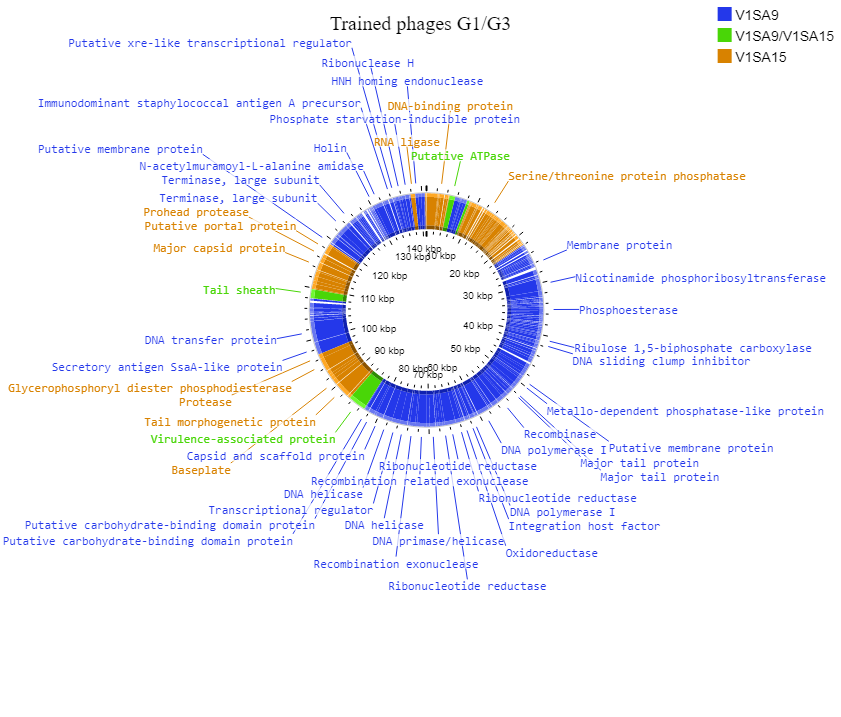
B**

**Supplementary Figure S2. Genomes of trained phages with prediction of origin of each gene.**

Genomes of phages isolated on S. epidermidis strains SE-E (A) and SE-G (B)
Predicted origin of each gene is indicated: V1SA9: blue, V1SA15: orange, mosaic genes: green or red.


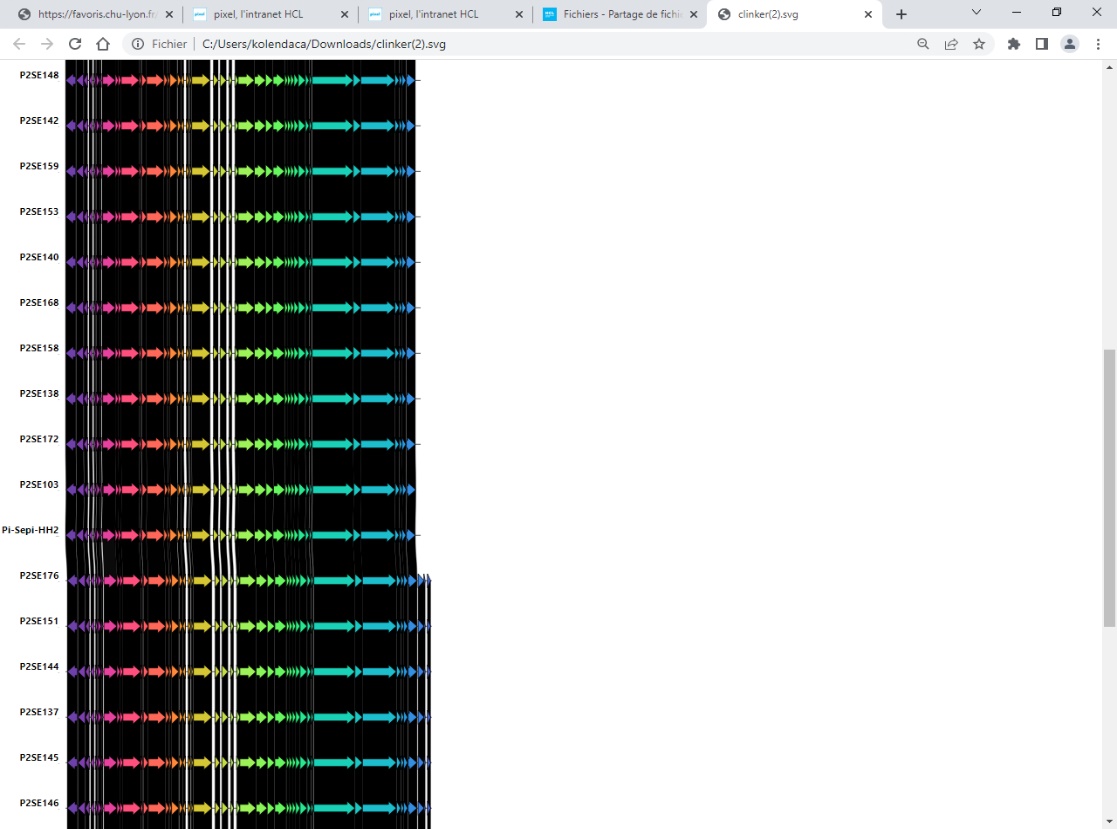

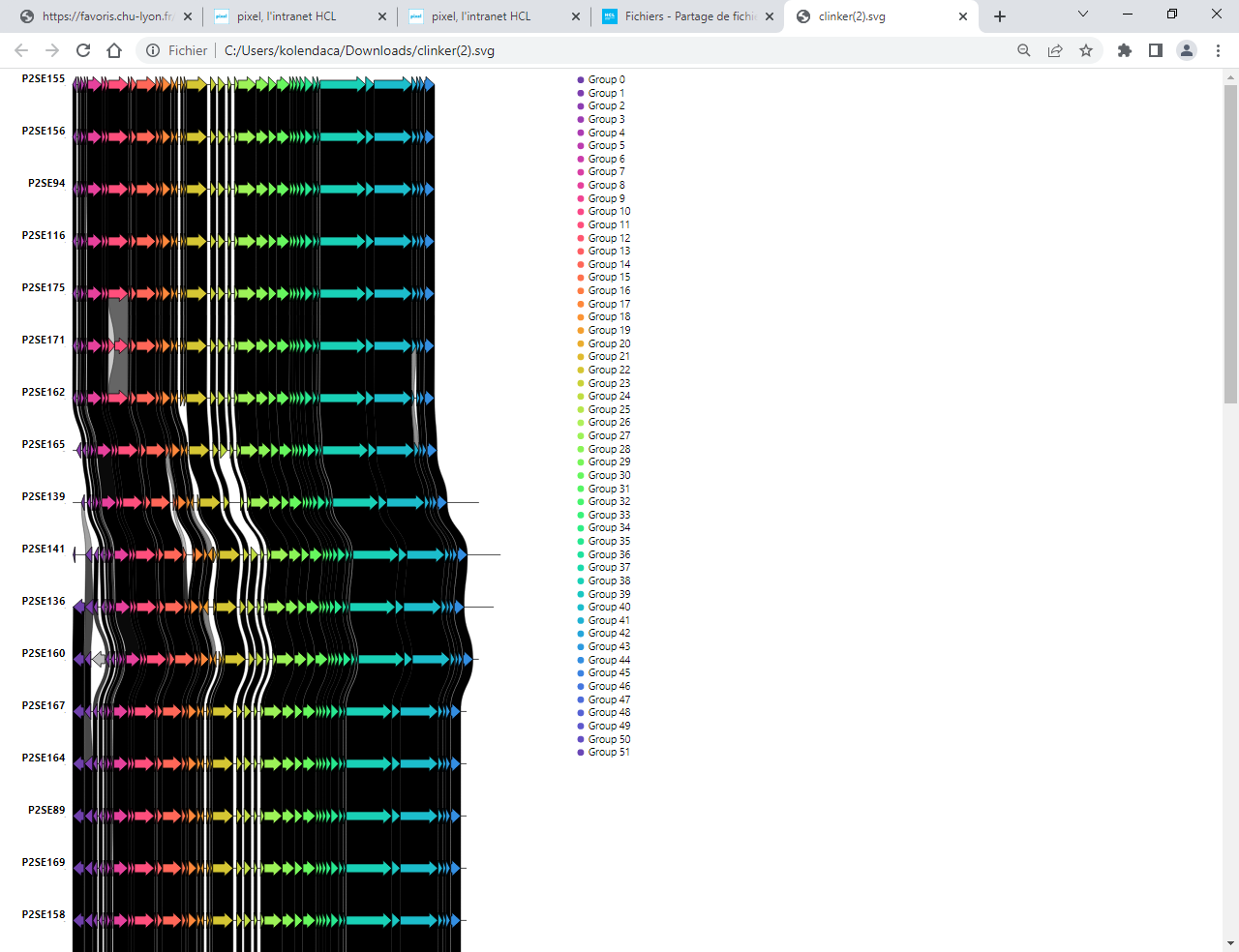

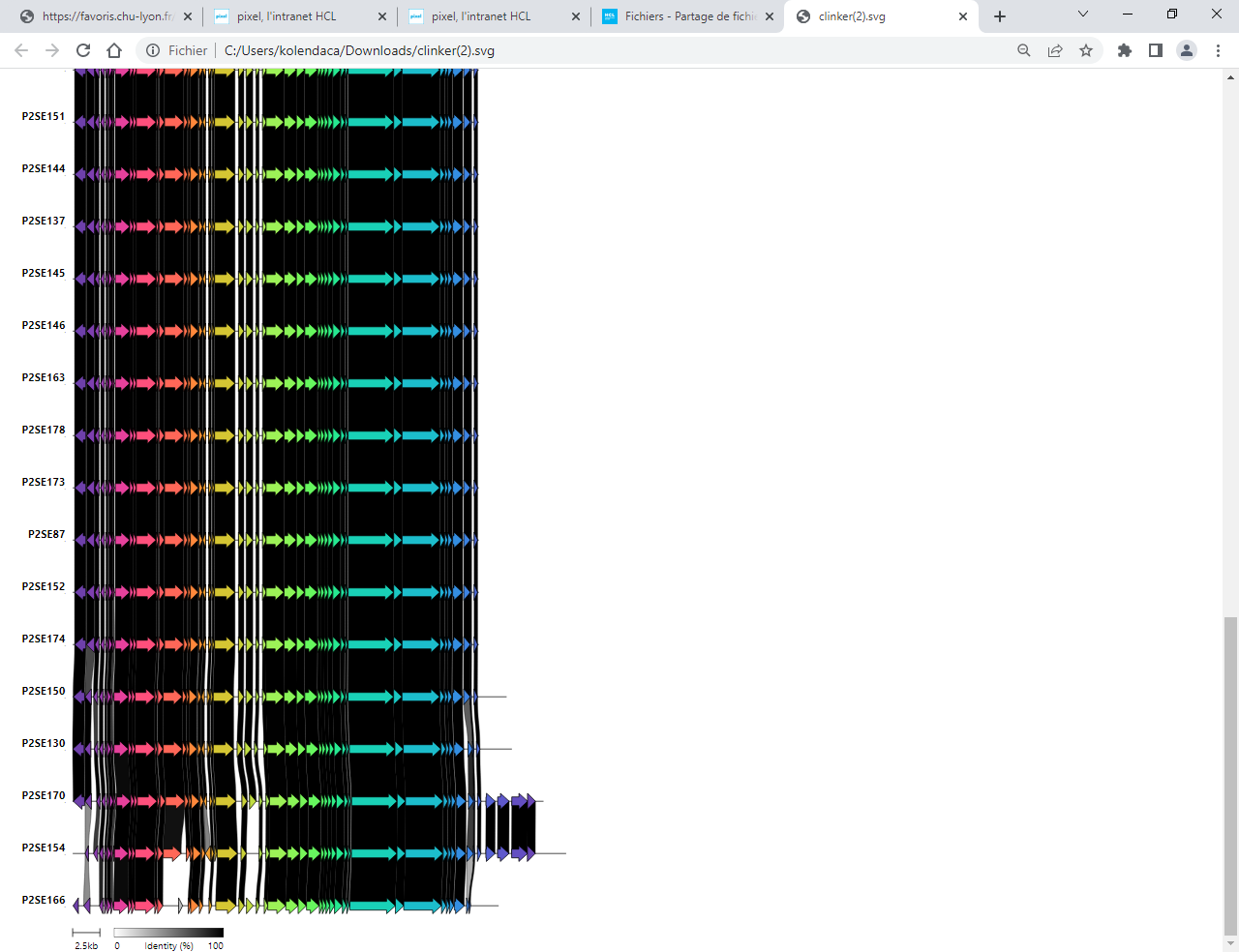

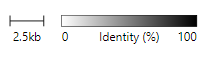


**Supplementary Figure S3. Alignment of prophage regions containing ST2-specific genes.**Each color indicates the presence of a different gene. Homology between genes of the prophage identified in each ST2 strain (in lines) is indicated by the shades of black using Clinker.
