# Supplementary material for: Potential of training of anti-Staphylococcus aureus therapeutic phages against Staphylococcus epidermidis multidrug-resistant isolates is restricted by inter- and intra-sequence type specificity
Source: mSystems. 2024 Sep 9;9(10):e00850-24. doi: 10.1128/msystems.00850-24 (PMC11494967; doi:10.1128/msystems.00850-24)
Supplement: Table S1 — Phage collection. [file msystems.00850-24-s0002.docx]

**Supplementary Table S1. Phage collection**

| Family/Genus | Phage | Isolation strain | Accession number | Genome size | Homology between phages (coverage/identity percentages) | | | | | |
| --- | --- | --- | --- | --- | --- | --- | --- | --- | --- | --- |
|  |  |  |  |  | V1SA09 | V1SA12 | V1SA15 | V1SA19 | V1SA20 | V1SA22 |
| *Herelleviridae*  *Kayvirus* | vB_SauM-V1SA09* | P2SA41 | OR602702 | 152,052 bp |  | 93/98 | 83/98 |  |  |  |
|  | vB_SauM-V1SA12* | P2SA40 | OR611155 | 151,107 bp | 93/98 |  | 82/97 |  |  |  |
|  | vB_SauM-V1SA15* | P2SA04 | OR602703 | 145,742 bp | 84/98 | 83/97 |  |  |  |  |
| *Herelleviridae*  *Silviavirus* | vB_SauM-V1SA19* | P2SA8 | ON814134 | 138,507 bp |  |  |  |  | 86/97 | 85/97 |
|  | vB_SauM-V1SA20* | P2SA58 | ON814135 | 136,886 bp |  |  |  | 86/97 |  | 94/99 |
|  | vB_SauM-V1SA22* | P2SA131 | ON814136 | 133,701 bp |  |  |  | 87/97 | 96/99 |  |

*Phage names are simplified throughout the manuscript (e.g. V1SA09 stands for vB_SauM-V1SA09) for clarity.
As coverage percentages between phages of different genera were low (< 25%), results of inter-genus pairwise comparisons were not indicated in this table.
