# Supplementary material for: Potential of training of anti-Staphylococcus aureus therapeutic phages against Staphylococcus epidermidis multidrug-resistant isolates is restricted by inter- and intra-sequence type specificity
Source: mSystems. 2024 Sep 9;9(10):e00850-24. doi: 10.1128/msystems.00850-24 (PMC11494967; doi:10.1128/msystems.00850-24)
Supplement: Table S4 — Genes specifically identified in ST2 strains. [file msystems.00850-24-s0005.docx]

**Supplementary Table S4. Genes specifically identified in ST2 strains.**

| **Gene number (example in strain P2SE103)** | **Annotation** | **InterPro prediction of protein function** | **HHpred hits** |
| --- | --- | --- | --- |
| 06990 | Peptidoglycan recognition family protein | Biological process: Peptidoglycan catabolic process  Molecular fonction: N-acetyl-L-alanine amidase activity  Family protein: None  Protein domain: N-acetylmuramoyl-L-alanine amidase domain | Endolysin, Autolysin,  N-acetylmuramoyl-L-alanine amidase |
| 07020 | Phage tail tape measure protein | Biological process: None  Molecular function: None  Family protein: None  Protein domain: Phage tail tape | Base plate, Tape measure protein |
| 07130 | DUF1672 / DUF3310 | Biological process: None  Molecular function: None  Family protein: Sav-like  Protein domain: None | Low propability hits only (< 60%) |
| 07165 | Hypothetical protein | Biological process: None  Molecular function: None  Family protein: None  Protein domain: None | Low propability hits only (< 40%) |
| 07180 | Hypothetical protein | Biological process: None  Molecular function: None  Family protein: None  Protein domain: None | Low propability hits only (< 40%) |
| 07185 | DUF 771 | Biological process: None  Molecular function: None  Family protein: None  Protein domain: None | Terminase, putative excisionase |
| 07190 | Helix turn helix transcriptional regulator | Biological process: None  Molecular function: DNA binding  Family protein: None  Protein domain: Cro/C1-type helix-turn-helix domain | Transcriptional activator, DNA binding protein, Repressor |
| 07195 | Helix turn helix transcriptional regulator | Biological process: None  Molecular function: DNA binding  Family protein: None  Protein domain: Cro/C1-type helix-turn-helix domain | Transcriptional activator, DNA binding protein, Repressor |
